# Supplementary material for: Phase Separation-Regulated Fungal Growth, Sexual Development, Adaptation and Synthetic Biology Applications
Source: J Fungi (Basel). 2025 Sep 17;11(9):680. doi: 10.3390/jof11090680 (PMC12470725; doi:10.3390/jof11090680)
Supplement: Supplementary file 1 [file jof-11-00680-s001.zip › Tab.S2.pdf]

Table S2. The result of the LLPS potential of WC-1 (UniProt ID:001371) predicted by the online LLPS prediction tools (ParSe, PSPredictor, PSP hunter and MolPhase).

**Method Name**	**Probability**
ParSe	β-turn propensity:0.989
PSPredictor	0.9845
PSP hunter	0.90368
MolPhase	0.999
