# Supplementary material for: Phase Separation-Regulated Fungal Growth, Sexual Development, Adaptation and Synthetic Biology Applications
Source: J Fungi (Basel). 2025 Sep 17;11(9):680. doi: 10.3390/jof11090680 (PMC12470725; doi:10.3390/jof11090680)
Supplement: Supplementary file 1 [file jof-11-00680-s001.zip › Tab.S1-ok.pdf]

Table S1. List of commonly used sequence-based prediction methods and tools of phase separation propensity.

Method Name	Principles	Datasets	web	Ref.
LARKS	Presence of low-complexity aromatic-rich kinked segments, Rosetta energy calculation	FUS, hnRNPA1 and NUP98 tandem repeats	_	[81]
PLAAC	Prion-like amino acid composition, a hidden Markov models trained on a defined set of prion proteins	_	plaac.wi.mit.edu	[75, 76]
CatGranule	RNA binding propensity, presence of structural disorder and residue patterns (RG/RGG and FG/GQ-rich sequences)	Foci-forming genes in the yeast proteome	_	[80, 81]
DDX4-like	Electrostatic interactions, regulation of PTM	Ddx4 variants	_	[81]
R + Y	Y/Y, Y/R, and R/R sidechain interactions, correlates well with the sequence length of FET family proteins, cSAT prediction	FET family proteins FUS, EWSR1, and TAF15, non-FET family proteins	_	[81]
PScore	the expected number of long-range, planar sp^2 pi–pi contacts	PDB structures with π interactions	pound.med.utoronto.ca/~JFKlab/#PScore	[77, 80,81]
PSPer	a 16-state hidden Markov-like model (HMM), representing four key domains, including RRM, PLD, Spacer and other structured domains, a machine learning predictor	RNA-binding proteins, prion proteins, disorder propensity, sequence composition and low complexity	bio2byte.be/b2btools/psp/	[77–82]
DeePhase	Random forest classifier, LM-word2vec, Presence of low-complexity regions, composition bias and structural disorder	LLPSDB and PDB	deephase.ch.cam.ac.uk/	[78, 80]
PSPredictor	The machine learning algorithm language model (LM)-word2vec, the componential and sequential information during the	LLPSDB，PSPredictor	pkumdl.cn:8000/PSPredictor/	[81]

	protein embedding stage, k-mer as 'words'			
PSAP	the sequence-based machine learning, a random forest algorithm trained on 55 biophysical features, including protein length, net charge, hydrophobicity, IDR fraction, secondary structure prediction, etc.	Predict new PPS proteins	github.com/Guido497/phase-separation	[81]
PhaSePred	PTM frequencies and immunofluorescence images	PhaSepDB, LLPSDB, and PhaSePro	-	[78]
LLPhyScore	Sequence-based physicochemical properties (net charge, hydropathy, aromaticity), structure-based features (DIR propensity, secondary structure predictions), **biophysical interaction parameters (π-π/cation-π potential, cross-β propensity)**	_	_	[80, 81]
ParSe	reciprocity between intramolecular forces that compact monomeric proteins and intermolecular interaction that drive phase separation, hydrodynamic size of monomeric proteins and β-turn-sequence propensity	PhaSePro and DisProt	stevewhitten.github.io/Parse_v2_FASTA.	[80]
Molphase	diverse physicochemical features, including high leucine content, glycine-rich regions, low solubility, intrinsic disorder, and cellular abundance, Manually curation of SG-associated proteins	phytobacterial type III effectors, DNA and RNA regulatory proteins in phytobacteria, PDB	_	[83]
dSCOPE	kAAP and PSSMs, machine learning, TPOT package	PS-driving regions and tumor mutations, as well as posttranslational modifications	_	[81]

FuzDrop	Enthalpic stabilization of the droplet state through physicochemical sequence features, Binary logistic regression model	PhaSepDB, PhaSePro, LLPSDB	fuzdrop.bio.unipd.it/predictor	[78]
PSPHunter	machine learning	driving residues in phase-separating proteins, FRAP, saturation measurements	psphunter.stemcellding.org	[80]
Droppler	The multi-head neural attention neural network, considered protein sequence features (embedding layers processing k-mer patterns and physical properties) and experimental conditions (temperature, salt, protein concentration, pH and presence of crowding agents)	PSPs and non-PSPs in LLPSDB	bitbucket.org/grogdrinker/droppler	[77, 78]

PSPs: phase-separating proteins; PLD: prion-like Domain; HMM: Hidden Markov model; LM: language model; GBDT: gradient boosting decision tree; PDB: protein data bank; RRM: RNA recognition motif; LCR: low complex region; IDR: intrinsically disordered region, PTM: post translational modification, HMM: hidden Markov-like model, SG: stress granule, TPOT: Tree-based Pipeline Optimization Tool, kAAP: k-spaced amino acid pairs, PSSMs: position-specific scoring matrices, “-”: none
